# Supplementary material for: A non canonical subtilase attenuates the transcriptional activation of defence responses in Arabidopsis thaliana
Source: eLife. 2016 Sep 29;5:e19755. doi: 10.7554/eLife.19755 (PMC5074803; doi:10.7554/eLife.19755)
Supplement: Supplementary file 1. — DOI: http://dx.doi.org/10.7554/eLife.19755.018 [file elife-19755-supp1.docx]

**Supplementary Table 1.** Oligonucleotide Primers Used in This Study

| **Name** | **Sequence** | **Polarity** | **Comments** | |
| --- | --- | --- | --- | --- |
| SBT5.2(a)attb1 | ggggacaagtttgtacaaaaaagcaggcttaatgaaaggcattacattcttc | (+) | SBT5.2(a) cloning in pDONR207/pBSDONRP1-P4 | |
| SBT5.2(b)attb1 | ggggacaagtttgtacaaaaaagcaggcttaatgaaaggcattacattcttca | (+) | SBT5.2(a) cloning in pDONR207/pBSDONRP1-P4 | |
| SBT5.2attb2 | ggggaccactttgtacaagaaagctgggtcgtttgtgcggctactctcg | (-) | SBT5.2(a) cloning in pDONR207 | |
| SBT5.2attb4 | ggggacaactttgtatagaaaagttgggtggtttgtgcggctactctc | (-) | SBT5.2(a) cloning in pBSDONRP1-P4 | |
| MYB30attb4r | ggggacaacttttctatacaaagttgcgatggtgaggcctccttgt | (+) | MYB30 cloning in pBSDONRP4r-P2 | |
| MYB30attb2 | ggggaccactttgtacaagaaagctgggtcatcagaagaaattagtgtt | (-) | MYB30 cloning in pBSDONRP4r-P2 | |
| MYB123attb4r | ggggacaacttttctatacaaagttgcgatgggaaagagagcaact | (+) | MYB123 cloning in pBSDONRP4r-P2 | |
| MYB123attb2 | ggggaccactttgtacaagaaagctgggtcatcaacaagtgaagtctcg | (-) | MYB123 cloning in pBSDONRP4r-P2 | |
| SBT5.2(a) ^N225A^-S | tctgccgtggaggctgcttcctactat | (+) | SBT5.2(a) PGS mutant | |
| SBT5.2(a) ^N225A^-AS | atagtaggaagcagcctccacggcaga | (-) | SBT5.2(a) PGS mutant | |
| SBT5.2(a) ^N363A^-S | ggtatacacttttcagccgttagtaaatctcct | (+) | SBT5.2(a) PGS mutant | |
| SBT5.2(a) ^N363A^-AS | aggagatttactaacggctgaaaagtgtatacc | (-) | SBT5.2(a) PGS mutant | |
| SBT5.2(a) ^N467A^-S | cttctcctacctcgcctcaaccaaagatcc | (+) | SBT5.2(a) PGS mutant | |
| SBT5.2(a) ^N467A^-AS | ggatctttggttgaggcgaggtaggagaag | (+) | SBT5.2(a) PGS mutant | |
| SBT5.2(a) ^N525A^-S | tgcatggactggagccgactcaagcatttc | (-) | SBT5.2(a) PGS mutant | |
| SBT5.2(a) ^N525A^-AS | gaaatgcttgagtcggctccagtccatgca | (+) | SBT5.2(a) PGS mutant | |
| SBT5.2(a) ^N636A^-S | tgttactatggatatgccgtaaccacaataaag | (-) | SBT5.2(a) PGS mutant | |
| SBT5.2(a) ^N636A^-AS | ctttattgtggttacggcatatccatagtaaca | (+) | SBT5.2(a) PGS mutant | |
| SBT5.2(a) ^N650A^-S | aagcttttccagaggcttttacttgccctg | (+) | SBT5.2(a) PGS mutant | |
| SBT5.2(a) ^N650A^-AS | cagggcaagtaaaagcctctggaaaagctt | (-) | SBT5.2(a) PGS mutant | |
| SBT5.2(a) ^N678A^-S | ctggattcaaaggagctggtagcaagacag | (+) | SBT5.2(a) PGS mutant | |
| SBT5.2(a) ^N678A^-S | ctgtcttgctaccagctcctttgaatccag | (-) | SBT5.2(a) PGS mutant | |
| SBT5.2^HtoA^-S | agggatgtcatcggtgccggttctcatgtgtc | (+) | SBT5.2 catalytic mutant | |
| SBT5.2^HtoA^-AS | gacacatgagaaccggcaccgatgacatccct | (-) | SBT5.2 catalytic mutant | |
| SBT5.1attb1 | ggggacaagtttgtacaaaaaagcaggcttaatgatgagatgcctcactatc | (+) | SBT5.1 cloning in pDONR207 | |
| SBT5.1attb2 | ggggaccactttgtacaagaaagctgggtcacgttcgctatcgttgtcgt | (-) | SBT5.1 cloning in pDONR207 | |
| VHA-a1attb1 | ggggacaagtttgtacaaaaaagcaggctcgatggaggaattcttagat | (+) | VHA-a1 cloning in pBSDONRP1-P4 | |
| VHA-a1attb4 | ggggacaactttgtatagaaaagttgggtggattaaagcgaaagagaa | (-) | VHA-a1 cloning in pBSDONRP1-P4 | |
| ARA6attb1 | ggggacaagtttgtacaaaaaagcaggctcgatgggatgtgcttcttct | (+) | Ara6 cloning in pBSDONRP1-P4 | |
| ARA6attb4 | ggggacaactttgtatagaaaagttgggtgtgacgaaggagcaggacg | (-) | Ara6 cloning in pBSDONRP1-P4 | |
| SYP21attb4r | ggggacaacttttctatacaaagttgcgatgagtttccaagatctc | (+) | SYP21 cloning in pBSDONRP4r-P2 | |
| SYP21attb2 | ggggaccactttgtacaagaaagctgggtcattagaccaacacaacgat | (-) | SYP21 cloning in pBSDONRP4r-P2 | |
| SYP61attb4r | ggggacaacttttctatacaaagttgcgatgtcctcagctcaagat | (+) | SYP61 cloning in pBSDONRP4r-P2 | |
| SYP61attb2 | ggggaccactttgtacaagaaagctgggtcattaggtcaagaagacaag | (-) | SYP61 cloning in pBSDONRP4r-P2 | |
| SBT5.2(b)^G2A^attb1 | ggggacaagtttgtacaaaaaagcaggctcgatggcatcagcttcctctgct | (+) | SBT5.2(b)^G2A^ cloning in pBSDONRP1-P4 | |
| SBT5.2(b)^162^attb1 | ggggacaagtttgtacaaaaaagcaggcttaatgtactataccacaagggatg | (+) | Truncated SBT5.2(b) cloning in pDONR207 | |
| SBT5.2(b)^342^attb1 | ggggacaagtttgtacaaaaaagcaggcttaatggtaaaagggaagattgtgt | (+) | Truncated SBT5.2(b) cloning in pDONR207 | |
| SBT5.1^405^attb1 | ggggacaagtttgtacaaaaaagcaggcttaatggtaaaagggaagattgtgt | (+) | Truncated SBT5.1 cloning in pDONR207 | |
| AtSBT5.2(a)-RT-S | gccatgaaaggcattacattct | (+) | SBT5.2(a) qRT-PCR | |
| AtSBT5.2(b)-RT-S | gccatgaaaggcattacattct | (+) | SBT5.2(b) qRT-PCR | |
| AtSBT5.2-RT-AS | gaagctgatcccatgtagacaa | (-) | SBT5.2 qRT-PCR | |
| SAND-S | aactctatgcagcatttgatccact | (+) | qRT-PCR | |
| SAND-AS | tgattgcatatctttatcgccatc | (-) | qRT-PCR | |
| CER2-S | agattcggttggcgagtattggtta | (+) | qRT-PCR | |
| CER2-AS | gcatacaaaatatcaacctccgaga | (-) | qRT-PCR | |
| FDH-S | cggttcttgtgctggttttt | (+) | qRT-PCR | |
| FDH-AS | gttcatcggagggcttgtaa | (-) | qRT-PCR | |
| SBT5.2^969^-AS | cctgtgttggtcatttgcac | (-) | 5’RACE 1^st^ PCR | |
| SBT5.2^1611^-AS | cctgatatgacgttatactgagaagc | (-) | 5’RACE 2^nd^ PCR | |
| SBT5.2 1482-AS | aaaatatgcaacagcagggg | (-) | 5’RACE 3^rd^ PCR | |
| \| \| \|  \| \| --- \| \| \|  \| \| --- \| \|  \| \| \| \| --- \| --- \| --- \| --- \| --- \| \|  \| \| --- \| --- \| --- \| --- \| --- \| --- \| --- \| \|  \|  \| \|  \|  \| | | | |  |
|  | | | |  |
